# PML: Not all about Tumor Suppression

**DOI:** 10.3389/fonc.2013.00200

**Published:** 2013-08-05

**Authors:** Natalia Martin-Martin, James D. Sutherland, Arkaitz Carracedo

**Affiliations:** ^1^CIC bioGUNE, Bizkaia Technology ParkDerio, Spain; ^2^Ikerbasque, Basque Foundation for ScienceBilbao, Spain; ^3^Biochemistry and Molecular Biology Department, University of the Basque Country (UPV/EHU)Bilbao, Spain

Since the discovery of Promyelocytic leukemia (PML), this protein has been associated with the pathogenesis of several hematopoietic malignancies and solid tumors. PML was first identified as part of a fusion oncoprotein, PML-RARα, responsible for the development of acute promyelocytic leukemia (APL) (1–4). The PML-RARα fusion protein not only alters PML function but also represses transcriptional activity mediated by RAR-RXR, thereby disrupting retinoid signaling, inhibiting myeloid differentiation and enhancing the survival and proliferation of early myeloid progenitors (5). Loss of PML in cancers from multiple origins underlines its tumor-suppressive role beyond leukemia (6).

Since PML seemed to be a key regulator underlying leukemia and other cancers, these initial findings motivated a series of studies aimed at ascertaining its regulatory cues and functions. It is now well established that PML is the building block of the PML-nuclear bodies (PML-NBs). PML functions as a protein scaffold and interaction partner for a growing number of factors that shuttle in and out of these structures in a highly regulated process (7–9).

## Expanding Cellular Functions of PML

Promyelocytic leukemia protein or PML exerts its anti-cancer role by modulating a number of pathways relevant to cancer biology. PML-NBs increase in number and size in response to DNA damage (10). The nuclear bodies co-localize with sites of single-stranded DNA recruitment and DNA repair. In turn, a number of DNA repair (e.g., MRE11, ATR, BLM, RAD) proteins dynamically localize to PML-NBs (11, 12). Furthermore, PML is an important regulator of both p53-dependent and p53-independent apoptotic pathways (13–15), accomplished by the activation of p53 or Fas, by the phosphorylation and activation of the checkpoint kinase CHK2 (16), or regulating mitochondrial-associated membrane (MAM) function (15). One of the PML anti-cancer and anti-proliferative functions is mediated by activation of the tumor suppressor p21, via transcriptional regulation by p53. Yang et al. demonstrated that big MAP kinase 1 (BMK1) interacts with PML suppressing p21 activation (17). BMK1 associates with PML and disrupts the interaction between PML and MDM2 (the major E3 ubiquitin ligase for p53), which leads to p53 stabilization. This effect induces an increase in tumor cell apoptosis *in vitro* and tumor regression *in vivo* (18). Additionally, PML suppresses neo-angiogenesis through the negative regulation of mTORC1 complex (19, 20).

Beyond the nucleus and the nuclear bodies, and perhaps the least studied aspect of PML, is the cytosolic localization and function of PML. Surprisingly, cytoplasmic PML mutants, with aberrant nuclear localization signal, function as a dominant negative, oncogenic forms of the tumor suppressor (21, 22). The Salomoni group (23, 24) reported that mutations in PML that re-localize the protein to the cytoplasm induced the recruitment and mis-localization of PML *wildtype* nuclear forms to this compartment, therefore reducing the number of PML-NBs. Cytoplasmic PML mutants inhibited p53 transcriptional, growth suppressive, and apoptotic functions (25). These data suggest that cytoplasmic expression of PML affects cell survival through inhibition of nuclear PML.

**Figure 1:**
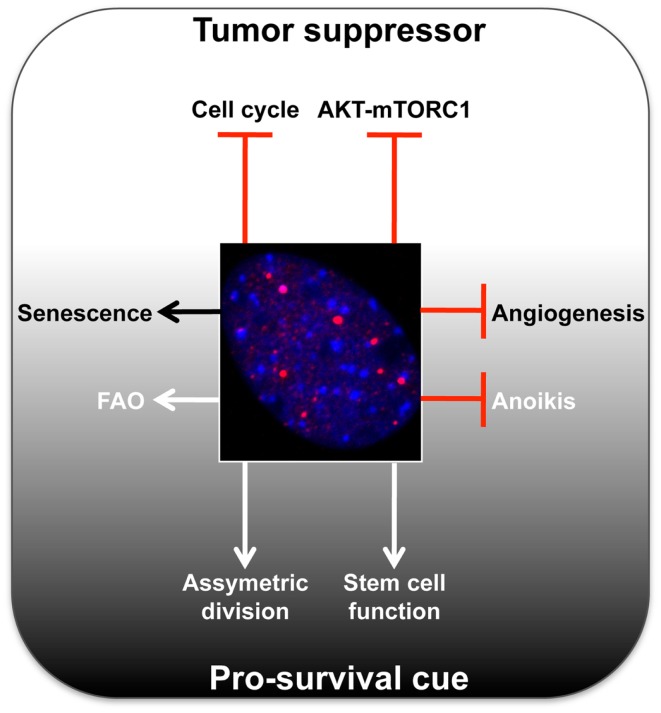
**Summary of main tumor-suppressive and pro-survival functions of PML**. A representative micrograph of PML immunofluorescence is shown in red, DAPI in blue.

In recent years, a growing body of work has revealed that PML may provide a selective advantage for tumor cells in certain settings (Figure 1), thus presenting PML as a therapeutic target. Is it possible that PML, in specific contexts (e.g., origin of tumor cell, microenvironments, or metabolic states) can provide a selective pro-survival benefit? In contrast to its usual perception as a classical tumor suppressor, below we will review the latest reports unveiling a potentially more sinister role for PML in cancer. To fully appreciate this novel role, it is important to first mention how the *PML* gene and protein are regulated.

## The Complex Regulation of PML

Promyelocytic leukemia is subject to extensive regulation at the transcriptional, post-transcriptional, and post-translational level. At the transcriptional level PML is induced by type I and II interferons (IFN), which cause an increase in both the size and number of PML-NBs (26). This is mediated through binding of IFN-stimulated transcription factors, known as signal transducers and activators of transcription (STATs) (27) and IFN-regulatory factors, such as IRF3 (28) and IRF8 (29), important mediators of myeloid cell differentiation. PML can also be transcriptionally and post-transcriptionally up-regulated by oncogenic Ras (30–34). At the post-transcriptional level, the PML gene can undergo alternative splicing which results in the production of >10 processed mRNAs and many resultant PML protein isoforms (35, 36). The different post-translational modifications have been recently comprehensively reviewed (37), and include phosphorylation, SUMOylation, and ubiquitylation. A more recent example is PML acetylation, which may play a role in apoptotic pathways (38). These modifications regulate the ability of PML to interact with various partners and confer stress- and signal-dependent regulation of PML or its binding proteins (37).

## Role of PML Beyond Tumor Suppression

An important study by the Pandolfi group (6) examined PML expression in a wide array of human cancers and revealed a surprising discovery: while PML protein expression was reduced or absent in numerous cancers (prostate, colon, breast, lung, lymphomas, CNS, germ cell tumors), PML mRNA was expressed in all tumors, rarely mutated and was not subject to loss of heterozygosity. Therefore, it was concluded that despite the presence of a functional gene, the PML protein is post-translationally degraded through proteasome-dependent mechanisms and its loss was generally associated with both tumor grade and progression.

In further studies on the role of PML, Ito et al. described that PML was highly expressed in cells from chronic myeloid leukemia (CML) patients, and in contrary to what had been described in solid tumors, loss of PML was predictive of favorable outcomes. Thus, PML expression was selected for and not against in CML (39). This unexpected finding was explained by a novel role of the PML protein in CML. PML was shown to be indispensable for quiescent leukemia-initiating cell (LIC) function; loss of PML resulted in both LIC and hematopoietic stem cell (HSC) depletion. It was also confirmed that treatment with As_2_O_3_ (arsenic trioxide or ATO), a drug that down-regulates PML through proteasomal-dependent degradation and that is currently used for the treatment of APL, was able to mimic the genetic loss of PML in mice (39). This finding pointed to a promising therapeutic application for this drug, specifically that destabilization of PML could eradicate LICs and provide a strong benefit for CML patients. The future development of additional, more selective PML-targeting drugs that promote its proteasomal degradation may be extremely helpful in the treatment of CML. These drugs may also find applications in other solid tumors where high levels of PML play a pathogenic role, as we will next discuss.

After this initial finding that PML may have a pro-survival role in CML, Ito et al. demonstrated that PML exerts its essential role in HSC maintenance through the regulation of fatty acid oxidation (FAO) (40, 41). HSCs remain in a quiescent state until environmental insults prompt them to enter the cell cycle, thus dividing and giving rise to multi-potent progenitors. Interestingly, FAO is essential to maintain this balance, under control of the peroxisome-proliferator activated receptor delta (PPARD). Moreover, PML exerts this essential role in HSC maintenance by acting upstream of PPAR signaling and FAO (41). Mechanistically, the PML-FAO PPARD pathway controls HSC asymmetric division. Loss of PML or PPARD, as well as mitochondrial FAO inhibition, resulted in symmetric commitment of HSC daughter cells, and concomitant failure to produce progenitor cells. Conversely, the pharmacological activation of PPAR increased asymmetric division and ensured the long-term self-renewal potential of HSCs.

From these studies, it seems clear that PML has two faces. PML can act as a classical tumor suppressor in many cancers, but in some cases can facilitate cancer cell survival. While studying PML expression in breast cancer biopsies, Carracedo et al. found that although PML protein expression was frequently low or undetectable in the majority of samples, a subset of breast cancer biopsies exhibited PML levels in tumor cells that were significantly higher than those observed in the normal epithelium (40). There was a significant correlation between PML protein and mRNA expression only in tumor (not stroma) cells. More importantly, high PML mRNA and protein expression was significantly associated with triple-negative breast cancer tumor subtype, high tumor grade, early tumor recurrence, and poor prognosis. This study further demonstrated that PML provides a selective advantage in response to metabolic stress triggered by conditions of loss of attachment in breast cancer cells. This was through regulation by PML of the same PPAR-FAO pathway, which stemmed from the PML-induced deacetylation and activation of the transcriptional cofactor PGC1A. PML expression in breast tumors was associated with a signature of activated PPAR signaling that controls FAO. This regulation is relevant to sustain ATP levels and potentially reduced NADP (42), when breast epithelial cells lose contact with the extracellular matrix. On the basis of these findings it is tempting to speculate that targeting both PML and FAO in triple-negative breast cancer tumors with combinations of ATO and other targeted therapies may present a novel approach to treating this tumor subtype.

Taken together, it makes sense that tumor cells would not select for the genomic loss of PML, as is seen with some classical tumor suppressor genes, since it would be irreversible and would prevent cells from utilizing the PML-mediated pro-survival or pro-self renewal pathways when challenged. Instead, transcriptional or post-translational regulation of PML expression and localization allows cancer cells to tune PML expression on the basis of the cellular context.

## Two-Faced Tumor Regulators: A Selective Club

Promyelocytic leukemia is not the only protein that has been described to have a dual role as a tumor suppressor and a pro-survival protein. TGFβ also exhibits a well-documented dual activity in cancer. The TGFβ signaling pathway negatively regulates cell growth, death, and immortalization (43). Thus, mutations or deletions in the TGFβ gene can lead to the onset of several tumors. TGFβ signaling also plays an important role as a positive regulator to modulate processes such as cell invasion, immune regulation, and microenvironment remodeling that can promote cancer progression, invasion, and tumor metastasis (43–45).

Notch is another example of dual activity in cancer regulation. It is well described that Notch activates signaling pathways that regulate cell division, growth, migration, differentiation, or death (46, 47). Similar to TGFβ, Notch activity is required for the physiological development of organisms and for the maintenance of adult tissues. However, it has been demonstrated that the deregulation of Notch signaling pathway or its pathological activation can induce certain types of tumors such as leukemia, breast, colon, skin, lung, or renal carcinomas (48–50).

Other examples of genes with dual activity in tumor biology are Toll-like receptors (TLRs) (51, 52), CD44 (53), sirtuins (54), or E-cadherin (55). In general, the activation of these proteins can promote pro-tumorigenic signaling and trigger the metastatic cascade, inducing proliferation, invasion, or apoptosis resistance. However, in different scenarios or signaling through specific networks, these proteins can have opposite effects on tumors, favoring tumor-suppressive responses.

From these and other examples, it seems clear that critical genes and their downstream pathways can also be involved in the transformation of normal healthy cells into cancerous, potentially malignant forms in multiple ways depending on the cell subpopulation, and the microenvironmental milieu. This idea is under intensive research and may play a decisive role in the search for novel therapeutic treatments aimed at specific cancer types and subtypes, a key concept in personalized medicine.

## Future Therapy: Combinatorial Approaches

These insights into the dual role of PML in tumorigenesis could lead to new therapeutic interventions. Twenty-five years of basic and clinical research have allowed most patients with APL to be definitively cured under ATO treatment combined with retinoic acid. ATO targets PML through oxidation-triggered disulfide bond formation and direct binding. This results in PML and PML-RARα SUMOylation, and its subsequent ubiquitylation and proteasome-mediated degradation (56, 57). As we have mentioned, in CML, a related but distinct cancer type, the PML protein is indispensable for quiescent LIC maintenance (39) through the regulation of FAO by PPAR signaling (41). Moreover, this is not the only case where PML plays a role as a pro-survival protein rather than a tumor suppressor. PML is highly expressed in a subset of breast cancers with worse prognosis and shorter time to recurrence (40). Therefore, the use of PML-targeting drugs that activate proteasomal degradation could be of remarkable interest in the treatment of CML and breast cancer. On the other hand, enhancing levels or activity of RNF4, an E3 ubiquitin ligase which is essential for ATO-induced PML degradation (56, 58), could also enhance the down-regulation of PML. With our new knowledge into the underlying mechanism of PML function, the pharmacological targeting of FAO, or the use of PPAR inhibitors in combination with low doses of ATO might exert a synergistic effect on triple-negative breast cancer tumors and possibly other solid tumors or leukemias.

In conclusion, PML has been revealed as friend and foe in cancer. More detailed studies are warranted in order to categorize diverse tumor types for these opposing activities of PML, and ultimately to explore the therapeutic potential of PML-targeting compounds, alone or in combination with drugs that target PML-linked pathways.
